# Variability of radiation doses of cardiac diagnostic imaging tests: the RADIO-EVINCI study (RADIationdOse subproject of the EVINCI study)

**DOI:** 10.1186/s12872-017-0474-9

**Published:** 2017-02-16

**Authors:** Clara Carpeggiani, Eugenio Picano, Marco Brambilla, Claudio Michelassi, Juhani Knuuti, Philipp Kauffman, S. Richard Underwood, Danilo Neglia, for the EVINCI Study Investigators

**Affiliations:** 1 CNR Institute of Clinical Physiology, via Moruzzi, Pisa, 1-56124 Italy; 2 Medical Physics Department, University Hospital ‘Maggiore della Carità, Corso Mazzini, 18, Novara, 28100 Italy; 3 University of Turku and Turku University Hospital, Kinakvarngatan 4-8, Åbo, 20520 Finland; 4 University Hospital Zurich, Rämistrasse 100, Zurich, 8091 Switzerland; 5 National Heart and Lung Institute, Imperial College London, London, UK; 6 Department of Nuclear Medicine, Royal Brompton and Harefield Hospitals, Sydney Street, London, SW3 6NP UK; 7 Fondazione Toscana G. Monasterio, via Moruzzi, 1, Pisa, Italy

**Keywords:** Medical imaging, Radiation dose exposure, Effective dose, CT

## Abstract

**Background:**

Patients with coronary artery disease can accumulate significant radiation dose through repeated exposures to coronary computed tomographic angiography, myocardial perfusion imaging with single photon emission computed tomography or positron emission tomography, and to invasive coronary angiography. Aim of the study was to audit radiation doses of coronary computed tomographic angiography, single photon emission computed tomography, positron emission tomography and invasive coronary angiography in patients enrolled in the prospective, randomized, multi-centre European study–EVINCI (Evaluation of Integrated Cardiac Imaging for the Detection and Characterization of Ischemic Heart Disease).

**Methods:**

We reviewed 1070 tests (476 coronary computed tomographic angiographies, 85 positron emission tomographies, 310 single photon emission computed tomographies, 199 invasive coronary angiographies) performed in 476 patients (mean age 60 ± 9 years, 60% males) enrolled in 12 centers of the EVINCI. The effective doses were calculated in milli-Sievert (mSv) as median, interquartile range (IQR) and coefficient of variation of the mean.

**Results:**

Coronary computed tomographic angiography (476 exams in 12 centers) median effective dose was 9.6 mSv (IQR = 13.2 mSv); single photon emission computed tomography (310 exams in 9 centers) effective dose was 9.3 (IQR = 2.8); positron emission tomography (85 in 3 centers) effective dose 1.8 (IQR = 1.6) and invasive coronary angiography (199 in 9 centers) effective dose 7.4 (IQR = 7.3). Inter-institutional variability was highest for invasive coronary angiography (100%) and coronary computed tomographic angiography (54%) and lowest for single photon emission computed tomography (20%). Intra-institutional variability was highest for invasive coronary angiography (121%) and coronary computed tomographic angiography (115%) and lowest for single photon emission computed tomography (14%).

**Conclusion:**

Coronary computed tomographic angiography and invasive coronary angiography doses vary substantially between and within centers. The variability in nuclear medicine procedures is substantially lower. The findings highlight the need to audit doses, to track cumulative exposures and to standardize doses for imaging techniques.

**Trial registration:**

The study protocol is available at https://www.clinicaltrials.gov/ (ClinicalTrials.gov Identifier: NCT00979199). Information provided on September 16, 2009.

## Background

Medical imaging is an important cause of exposure to radiation, and cardiac imaging in particular has contributed greatly to its recent increases, especially in patients with known or suspected coronary artery disease (CAD) [1, 2], such as those involved in the EVINCI study, which was designed to identify the optimal noninvasive strategy to diagnose significant CAD [3]. Radiation dose is an important component of the ratio of benefit to risk for any given combination of diagnostic tests being related to the possible long term adverse risks [4, 5]. It has therefore been proposed that the total effective dose (E) per episode of care should be documented so that lifetime exposure can be estimated [6–8]. Estimated cumulative E is a potential safety metric for patients with common clinical conditions [9], and it is especially relevant in patients with known or suspected CAD, whose cumulative radiation exposure has risen 4-fold in the last 40 years [10] because of increases in the use of imaging tests.

The aim of this study was to evaluate the E of patients with intermediate likelihood of CAD enrolled in the EVINCI trial, which evaluated the accuracy of a combination of a non-invasive purely anatomical imaging as coronary computed tomographic angiography (CCTA) with a non-invasive functional approach as myocardial perfusion imaging with single photon emission computed tomography (SPECT) and positron emission tomography (PET) for the identification of anatomically and functionally significant CAD, as defined by invasive coronary angiography (ICA) [3].

## Methods

### Study population

The study included 476 consecutive patients (mean age 60 ± 9 years, 286 males) prospectively enrolled in the EVINCI trial, which studied 697 patients between January 1, 2009 and May 20, 2012, from 12 different European institutions [3]. The study protocol is available at http://www.clinicaltrials.gov (NCT00979199). The main aim of the EVINCI study was to identify the optimal strategy for noninvasive detection of CAD. By inclusion criteria, all patients had intermediate likelihood of CAD and no previous myocardial infarction, percutaneous coronary intervention or coronary artery bypass grafting. Major exclusion criteria were: age < 30 > 75 years; left ventricular dysfunction (EF < 35%); low or high (< = 20%, > = 90%) probability of CAD; persistent atrial fibrillation or advanced AV Block; asthma or chronic treatment with aminophylline; <6 months cerebral ischemic attack; peripheral vascular disease; cancer; severe hypertension; congenital heart disease; significant valvular disease; inability to provide an informed consent. The radiation sub-study included only patients in which the radiation dose was recorded at the end of at least one test and sent to the core lab. The correct recording of dose was obtained in 476 of the initial enrolled 697 patients; of these, 78 were drop-outs and 619 had an imaging evaluation; of these 619, 476 (77%) had the radiation dose information and represent the population of the present study. ICA conducted at the same time as percutaneous coronary intervention was excluded because of the difficulty of separating the dose of each procedure. The characteristics of the patients are reported in Table 1.

**Table 1:** Patient characteristics

Number	476
Age, yrs, mean ± SD	60 ± 9
Male, no. (%)	286 (60)
≥1 vessel disease, no.(%)	110 (23)
Hypertension, no. (%)	298 (62)
Diabetes, no. (%)	120 (25)
Hypercholesterolaemia	284 (59)
Smoking, no. (%)	120 (25)
Family history of CAD, no. (%)	166 (34)
BMI, kg/m2, mean ± SD	27.90 ± 4.7
No risk factors	42 (9)
Angina	
Typical	126 (26)
Atypical	292 (61)
ECG	
Abnormal	97 (20)
Normal	360 (75)
Therapy	397 (83)

*BMI* body mass index

### Dose estimates

To evaluate the radiation exposure for each imaging procedure, we obtained estimates of typical E (assessed in mSv).

#### Nuclear medicine

The E from nuclear medicine was estimated from the radiopharmaceutical used and its activity. Coefficients relating E to administered activity were obtained from the addenda to International Commission on Radiologic Protection Publication 53 [11, 12].

#### CCTA

The E for CCTA to 70 kg patients was obtained by the use of a conversion factor between E and dose length product (DLP) in cardiac examinations of 0.026 mSv/milligray.(mGy).cm which takes into account ICRP 103 weighting factors and is valid at 120 keV [13]. E for a patient of weight W was computed as:

$$ \mathrm{E}\left(\mathrm{mSv}\right)=\mathrm{D}\mathrm{L}\mathrm{P}\left(\mathrm{mGy}.\mathrm{cm}\right)\times 0.026\left(\mathrm{mSv}.{\mathrm{mGy}}^{-1}.{\mathrm{cm}}^{-1}\right)\times R\left(\mathrm{W}\right) $$

where R(W) is a dimensionless patient weight correction factor reported by Huda et al. [14] as:

$$ \mathrm{R}\left(\mathrm{W}\right)=1.73-1.33\mathrm{E}-2\mathrm{W}+4.04\mathrm{E}-5{\mathrm{W}}^2 $$

with W expressed in kg. Because R(W) is applied to all irradiated organs in each CCTA scan, Es can be adjusted for patient size by using the same scaling factor as applied to organ doses.

#### ICA

Finally the E for *ICA* was obtained by the use of the National Radiological Protection Board (NPRB) model which assumes a conversion factor between E and dose-area product, measured in Gy.cm^2^ of 0.18 mSv.Gy^−1^.cm^−2^ [15].

### Statistical analysis

Data were described using median and interquartile range (IQR) for non-normal distributions and mean, standard deviation and coefficient of variation for normal distributions. Inter-institutional variability was expressed as the coefficient of variation of mean E. Intra-institutional variability was expressed as the average coefficient of variation of mean E in each center. Comparison between groups was performed using Fisher’s exact test for categorical variables and Mann-Whitney U test or Kruskal-Wallis for non-normally distributed continuous variables with two groups or more than two groups, respectively. Hierarchical regression model adjusted for age, sex and BMI was used to evaluate the possible influence on E of type of equipment for each procedure and of protocol for SPECT. Box and whiskers plots were used to provide a univariate graphical representation of E obtained in the different centers for the different imaging techniques. Outliers and extremes were defined as points higher than the value of the 75th percentile plus 1.5 or 3 times the interquartile distance, or lower than the value of the 25th percentile minus 1.5 or 3 times the interquartile distance, respectively. All statistical analyses were carried out with Statistica software, version 6.0 (Statsoft) using a two-sided type I error rate of 0.05.

## Results

Radiation dose was available in 1070 imaging tests performed in 476 patients (286 males, 60%). Table 2 shows the scanner types in use in the different centers. The imaging procedure radiation dose distribution is shown in Table 3.

**Table 2:** Scanner manufactures and type

Procedure	Scanner Manufactures	Type	Centers
*CCTA*			
	General Electric	Light Speed VCT	4
	Philips	Brilliance 64	4
	Siemens	Somatom Definition DS	1
	General Electric	PET/CT Discovery 690	1
	Siemens	PET/CT Biograph 16	1
	Siemens	Somatom Definition Flash	1
*SPECT*			
	General Electric	Discovery NM 530C	1
	Picker	Axis	1
	General Electric	Millenium VG	2
	General Electric	Infinia Hawkey 4	3
	Philips	Brightview XCT	1
	Siemens	Ecam	1
*ICA*			
	General Electric	Integris Allura 9 biplane	1
	Siemens	Axiom Artis	2
	Philips	Allura XPER FD10	3
	General Electric	Innova 2100	1
*PET*			
	General Electric	Discovery 690	3

*CCTA* coronary computed tomographic angiography, *SPECT* single photon emission computed tomography, *ICA* invasive coronary angiography, *PET* positron emission tomography

**Table 3:** Effective dose estimates for the different centers

Center	CCTA (mSv)			SPECT (mSv)			PET (mSv)			ICA (mSv)		
	*CV%*	*N*	*Mean ± SD* *Median, IQR*	*CV%*	*N*	*Mean ± SD* *Median, IQR*	*CV%*	*N*	*Mean ± SD* *Median, IQR*	*CV%*	*N*	*Mean ± SD* *Median, IQR*
*1*	26	64	*9.9 ± 2.6* *9.6,1.1*				22	57	1.8 ± 0.4 2.0, 0.2	121	49	9.2 ± 11.2 6.3, 6.2
*2*	58	71	3.1 ± 1.8 2.5, 1.4	14	71	10.1 ± 1.4 9.4, 2.8		1	1.0	102	15	18.7 ± 19.1 12.1, 24.5
*3*	54	3	12.2 ± 6.6 15.6, NC									
*4*	38	42	14.5 ± 5.5 12.9, 8.1	17	43	14.0 ± 2.4 14.2, 3.8				65	30	5.5 ± 3.6 4.5, 4.3
*5*	92	34	6.7 ± 6.2 4.5, 0.9	33	25	9.1 ± 3.0 9.2, 5.1	32	4	2.0 ± 0.6 2.0, 0.9			
*6*	20	15	13.0 ± 2.6 13.7, 4.0	14	16	9.5 ± 1.3 9.2, 0.7						
*7*	27	17	20.6 ± 5.5 22.7, 7.7	19	17	11.7 ± 2.2 12.5, 0				63	4	10.7 ± 7.0 11.5,13.3
*8*	19	50	20.9 ± 3.9 20.6, 4.2	15	50	10.1 ± 1.5 9.3, 1.0				69	31	13.6 ± 9.4 11.1, 7.9
*9*	53	36	5.1 ± 2.7 4.6, 1.1							59	4	2.8 ± 1.6 2.8, 2.9
*10*	44	44	5.7 ± 2.5 5.7, 4.2	30	36	8.0 ± 2.4 8.6, 4.1				60	36	6.1 ± 3.7 4.8, 4.7
*11*	49	86	17.6 ± 8.6 17.4, 8.5	23	44	7.9 ± 1.8 8.7, 1.2	25	23	2.3 ± 0.6 2.4, 0.7	50	26	10.9 ± 5.4 10.2, 6.9
*12*	115	14	6.7 ± 7.7 4.0, 2.1	21	8	8.4 ± 1.8 9.1, 1.1				54	4	9.3 ± 5.0 10.3, 9.0
*Tot*	54	476	11.2 ± 8.1 9.5, 13.2	20	310	10.0 ± 2.7 9.3, 2.8	50	85	1.4 ± 0.7 1.8, 1.6	100	199	9.64 ± 9.7 7.4, 7.3

CCTA, SPECT, PET, ICA as in Table 2; *CV* average coefficient of variation of E in each center, *IQR* interquartile range, *N* number of patients

Four hundred seventy-six CCTA were obtained in 12 centers, 394 myocardial perfusion imagings, of which 85 PET in 3 centers and 310 SPECT in 9 centers (1 patient performed both); 199 ICA in 9 centers.

**Fig. 1:**
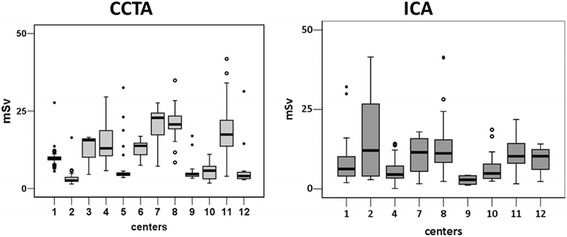
Coronary angiography effective dose. Distribution of median (interquartile range) coronary angiography effective dose (mSv) obtained by coronary computed tomographic angiography (CCTA) (*left panel*) and by invasive coronary angiography (ICA) (*right panel*). The whiskers show the minimum and maximum observed values. mSv: milliSievert

**Fig. 2:**
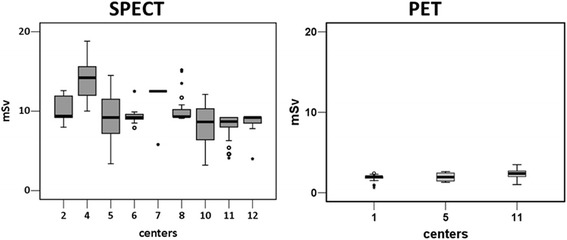
Nuclear medicine effective dose. Distribution of median (interquartile range) myocardial perfusion effective dose (mSv) obtained by single photon emission computed tomography (SPECT) (*left panel*) and positron emission tomography (PET) (*right panel*). The whiskers show the minimum and maximum observed values. mSv: milliSievert

Estimates of E for each of the procedures (CCTA, SPECT, PET, ICA ) are shown: the mean highest dose was obtained for CCTA and the lowest for PET. Intra-institutional variability was highest for ICA (121%) and for CCTA (115%) and lowest for SPECT (14%). Inter-institutional variability was highest for ICA (100%) and for CCTA (54%) and lowest for SPECT (20% ) (Table 3). The variability for each test is shown in Figs. 1 and 2. The results of hierarchical regression model adjusted for age, gender and BMI are presented in Table 4.

**Table 4:** Multivariable regression analysis

Factor		*B*	95% CI	Standard error	R square change	*P*
*CCTA*						
Model 1: age		0,17	0,085;0,255	0,043	0,040	<0,001
Model 2: equipment		−0,55	−1,02; −0,088	0,24	0,012	0,020
*SPECT*						
Model 1:	BMI	0,199	0,125;0,273	0,038	0,163	<0,001
	sex	−1,151	−1,836; −0,465	0,348	0,163	0,001
Model 2: equipment		−0,418	−0,*605*; −0,231	0,095	0,070	<0,001
Model 3: protocol		2,129	1,545; 2,713	0,296	0,150	<0,001
*ICA*						
Model 1:	BMI	1,225	0,751;1,700	0,240	0,191	<0,001
	sex	7,435	2,939;11,931	2,278	0,191	0,001
Model 2: equipment		−4,276	−6,137; −2,415	0,943	0,087	<0,001

Model 1: Predictors BMI, age, sex; Model 2: Predictors BMI, age, sex, type of equipment; Model 3: Predictors BMI, age, sex, type of equipment, type of protocol (1 or 2 days protocol)

The type of equipment was a significant independent predictor of E for each imaging procedure although the percent of E variability accounted for by this predictor was only 1,2% for CCTA and 7 and 8,7% for SPECT and ICA respectively. When the type of SPECT protocol was added to the regression model the predictive power increased of 15%.

E was significantly different between males and females for ICA (15 ± 15 versus 9 ± 13, *P* = 0.001) and SPECT (11 ± 3 versus 10 ± 3, *P* = 0.011). Mean age was 60 ± 9 with centers ranging from 65 ± 6 to 54 ± 8 years (*P* < 0.001); its predictor effect was significant for SPECT and ICA E. Mean body mass index was 28 ± 4, with centers ranging from 25 ± 5 to 29 ± 4 (*P* = 0.007).

There was a significant difference of radiation dose between centers even using the same type of CCTA scanner (4 centers, *P* < 0.001 and 4 centers *P* < 0.001). There were also differences between centers using the same SPECT camera (3 centers, *P* < 0.001) and ICA manufacturer (3 centers, *P* = 0.017 and 2 centers, *P* = 0.047) (Table 2).

## Discussion

We have found that in patients evaluated for suspected CAD, radiation doses received from diagnostic interventions varied substantially within and between centers and the variability was highest for ICA and CCTA and lower for SPECT and PET, even between centers using the same equipment.

The observed great variability of E for each examination can recognize different factors: patient-related, technology-related and operator-related. Of interest, we found substantial inter-center differences even in centers using the same technology, suggesting that factors related to the operator, as experience, adherence to the best practice and awareness can play a major role in optimizing dose delivery for any given study. This can certainly have to do with increasing experience of the operators, but also–at least equally important–with the individual practitioner awareness and concern for radioprotection issues. In fact, radiation awareness regarding doses employed in commonly used examinations can be low even in very experienced cardiologists or radiologists or nuclear cardiologists, in high volume laboratories [16, 17]. The dose optimization is not only driven by the number of examinations you did in the past, but by the attention paid to radioprotection in the working habitat you live in. The large intra-site variability of ED could also be viewed as a good thing, since a personalized application of the ALARA-principle sometimes should lead to a large variation in patient dose.

Another potential, hidden source of variability is underreporting of doses by recruiting centers. It is of concern that about 1 out of 4 studies did not include a dose report, in spite of the fact that the radiation assessment was a clearly specified subproject of the study, and all centers had long-standing experience and reputation in cardiac imaging. According to the ESC position statement on medical radiation in cardiology, “due to the numerous sources of variability there is no clear threshold between acceptable and unacceptable exposure for any given examination, but the dose that is not even considered or reported is certainly unacceptable” [18].

The regression model showed that the type of equipment was a significant and independent predictor of E mainly for ICA although the percent of E variability accounted for by this predictor was modest. Relative to perfusion imaging the type of technology used was important, with PET associated with lower doses than SPECT, as well as SPECT type of protocol. Different equipment has different dose-reduction potential, particularly the ones developed in recent years and also different costs. The physiological heterogeneity due to uneven distribution of health care resources may have been magnified in recent years by the economic crisis, which has led in Europe to a reduction in the turnover of imaging equipment and failure to invest in lower dose equipment to reduce medical radiation exposure [19].

The dose values that we found are by and large consistent with the recent data reported in the literature. In the prospective, multicenter, US-based PROMISE trial, Douglas et al. [20] reported a median dose around 10 mSv for CCTA and nuclear stress testing, with similar dose variability as reported in the present study. In fact, the two studies share some important similarities in design eventually affecting the similarities in radiation dose results. Both EVINCI and PROMISE included minimal requirements for all testing methods, and observed similar variation across sites likely due to the expected differences in operator awareness of radioprotection, experience, equipment, and image-acquisition protocols [3, 20]. Our data are also consistent with the INCAPS trial, showing a wide inter-institutional dose variability in stress perfusion imaging, with the lowest doses in nuclear cardiology practice achieved when the radiation best practices are followed [21].

The culture and technologies are evolving in cardiology and rapidly progressing towards the goal of achieving the same imaging information with less dose. With nuclear cardiology, this is best achieved with stress-only protocol, technetium-based tracers, and new SPECT detectors which reduce the dose with preserved image quality which in EVINCI study were not yet mostly in use. For myocardial perfusion imaging PET produces at least comparable diagnostic information compared with SPECT but with almost 5-fold reduction in dose. There are however variations in dose between PET centers arising mainly from the use of different radiopharmaceuticals, with rubidium-82 having somewhat higher dose than 15O-water or 13N-ammonia [11, 12]. Whether these differences in dose between PET and SPECT justify the higher cost requires further analysis.

In CCTA, new breakthrough technologies allow coronary imaging with sub-mSv doses [22]. In invasive cardiology, zero-fluoroscopy navigation techniques in electrophysiology and dose-sparing technology in fluoroscopy coupled with better operator training and awareness will slash by 50% or more the dose exposure associated with common procedures [23]. It is the responsibility of the operators to use all the available resources to obtain the optimal radiation dose management which means that it is essential to develop a culture of radiation safety. radiation safety. It is expected that the dose burden for imaging cardiology patients will be substantially reduced in next years. The combined effects of technology evolution in imaging, greater awareness of radiation risk in cardiology and, at least in Europe, changes in legislation forcing laboratories to communicate in writing the dose administered during the examination, starting February 2018, will likely change the current heterogeneous dosimetric landscape for the better in the near future.

An improved culture of radioprotection will enormously benefit not only the patients, but also the doctors, who will have their professional life longer and healthier if the principles of justification, optimization and protection are implemented, especially in the invasive imaging laboratories [18].

### Study limitations

The study has some methodological limitations. Reference doses cannot be measured precisely nor are they patient-specific [7, 24]. E is an estimate designed to provide a sex-averaged dose for a reference subject and it may not apply to individual patients [8]. It relies on assumptions of the radiation sensitivity of organs and tissues, imaging techniques and protocols, and, in the case of radionuclide imaging, radiopharmaceutical activity, half-life, distribution and elimination kinetics [25–27]. Moreover the E for CCTA is estimated by multiplying the DLP with a conversion factor which affects the E values reported in the literature [28]. We applied a value of 0.026 mSv mGy^−1^ cm^−1^ since this value was likely to be more accurate for estimation of radiation dose associated with CCTA compared to the ones usually assumed for chest CT (0.014 or 0.017 mSv mGy^−1^ cm^−1^) [13, 28]. Despite its use raises controversy, E remains the only available measure of the potential biologic effects of different types of radiation. The description of imaging protocol, which could be helpful to understand the large variability in E, was not included in the EVINCI radiation substudy database. Unfortunately we do not have information on the operators years of experience to be included in the regression model to test its predictive power for the different procedures.

## Conclusions

Although most of cardiac imaging tests are performed in elderly patients, the cardiac patients are today the patients living longer because of advances in disease prevention and management. This means that we should pay attention to the long-term side effects of our interventions, including imaging procedures using ionizing radiation. These forms of imaging have improved the diagnosis and treatment of numerous, if not all, cardiac diseases. At the same time, some of them expose patients to ionizing radiation, which has long-term side effects. The contemporary patient who undergoes interventional procedures may receive a median effective dose of 60 mSv, with 1 out of 4 patients exceeding 100 mSv [1]. In the USA, high doses from medical imaging procedures (>20 mSv/year) are experienced by 2% of the population [2]. Since a significant part of our imaging examinations may not be justified or optimized [29] our findings encourage greater awareness of radiation doses and help us to ensure the best possible ratio of benefit to risk for our patients, taking into account even the possible long term, adverse effects of radiation use.

Special care to justification and optimization should be taken in subgroups particularly vulnerable to radiation effects, such as children and women. For any given radiation exposure, the cancer risk is 2-to-ICA, coronary angioplasty and CCTA by breast, a highly radiosensitive organ in women [17]. The single exposure to the individual examination may be negligible, but all doses add-up in determining the lifetime exposure and individual risk. Exam adds to exam, dose to dose and risk to risk, and therefore the entire radiological dosimetric record of the patient should be considered in determining the individual risk, especially considering age and gender, with more restrictive criteria applying to younger and female patients, and those who received already a significant exposure in the past.

In conclusion the findings highlight the need to audit doses in cardiac imaging labs, to track cumulative exposures and to standardize doses for each technique. This high priority scientific and clinical need is also propelled by the European radiation protection legislation which was recently updated, and now requires monitoring of radiation exposure of patients [30]. All member states are forced to transpose the Directive into national legislation and to implement its requirements by 2018.

## Abbreviations

BMI: Body mass index
CAD: Coronary artery disease
CCTA: Coronary computed tomographic angiography
CV: Coefficient of variation
DLP: Dose length product
E: Effective dose
EVINCI: Evaluation of Integrated Cardiac Imaging for the Detection and Characterization of Ischemic Heart Disease
ICA: Invasive coronary angiography
IQR: Interquartile range
mGy: milligray
mSv: milli-Sievert
PET: Positron emission tomography
SPECT: Single photon emission computed tomography
W: Weight
